# CXCR4 and CXCR7 Inhibition Ameliorates the Formation of Platelet–Neutrophil Complexes and Neutrophil Extracellular Traps through Adora2b Signaling

**DOI:** 10.3390/ijms222413576

**Published:** 2021-12-17

**Authors:** Kristian-Christos Ngamsri, Rizki A. Putri, Christoph Jans, Katharina Schindler, Anika Fuhr, Yi Zhang, Jutta Gamper-Tsigaras, Sabrina Ehnert, Franziska M. Konrad

**Affiliations:** 1 Department of Anesthesiology and Intensive Care Medicine, University Hospital of Tübingen, Hoppe-Seyler-Str. 3, D-72076 Tübingen, Germany; kristian.ngamsri@med.uni-tuebingen.de (K.-C.N.); rizkianindyaputri95@gmail.com (R.A.P.); Christoph.jans@uni-tuebingen.de (C.J.); k.schindler1@web.de (K.S.); Anika.fuhr@uni-tuebingen.de (A.F.); yi.zhang.cn23@gmail.com (Y.Z.); jutta.gamper-tsigaras@klinikum.uni-tuebingen.de (J.G.-T.); 2 Siegfried Weller Research Institute, BG Trauma Center Tübingen, Department of Trauma and Reconstructive Surgery, University of Tübingen, Schnarrenbergstr. 95, D-72076 Tübingen, Germany; sabrina.ehnert@med.uni-tuebingen.de

**Keywords:** acute inflammation, PMNs, thrombocytes, PNCs, NETosis, SDF-1 receptors, acute peritonitis, sepsis

## Abstract

Peritonitis and peritonitis-associated sepsis are characterized by an increased formation of platelet–neutrophil complexes (PNCs), which contribute to an excessive migration of polymorphonuclear neutrophils (PMN) into the inflamed tissue. An important neutrophilic mechanism to capture and kill invading pathogens is the formation of neutrophil extracellular traps (NETs). Formation of PNCs and NETs are essential to eliminate pathogens, but also lead to aggravated tissue damage. The chemokine receptors CXCR4 and CXCR7 on platelets and PMNs have been shown to play a pivotal role in inflammation. Thereby, CXCR4 and CXCR7 were linked with functional adenosine A2B receptor (Adora2b) signaling. We evaluated the effects of selective CXCR4 and CXCR7 inhibition on PNCs and NETs in zymosan- and fecal-induced sepsis. We determined the formation of PNCs in the blood and, in addition, their infiltration into various organs in wild-type and Adora2b−/− mice by flow cytometry and histological methods. Further, we evaluated NET formation in both mouse lines and the impact of Adora2b signaling on it. We hypothesized that the protective effects of CXCR4 and CXCR7 antagonism on PNC and NET formation are linked with Adora2b signaling. We observed an elevated CXCR4 and CXCR7 expression in circulating platelets and PMNs during acute inflammation. Specific CXCR4 and CXCR7 inhibition reduced PNC formation in the blood, respectively, in the peritoneal, lung, and liver tissue in wild-type mice, while no protective anti-inflammatory effects were observed in Adora2b−/− animals. In vitro, CXCR4 and CXCR7 antagonism dampened PNC and NET formation with human platelets and PMNs, confirming our in vivo data. In conclusion, our study reveals new protective aspects of the pharmacological modulation of CXCR4 and CXCR7 on PNC and NET formation during acute inflammation.

## 1. Introduction

Peritonitis and peritonitis-related sepsis are characterized by an excessive immune response leading to high mortality [1]. However, peritonitis and peritonitis-related sepsis are mainly triggered by microbial invasion into various tissues or body fluids (1). Despite advances in supportive care and intense basic research, new specific therapies remain elusive for septic patients [2]. During acute inflammation, circulating inflammatory cytokines activate platelets and polymorphonuclear neutrophils (PMNs), resulting in the formation of platelet–neutrophil complexes (PNCs). PNCs promote the migration of PMNs into the inflamed tissue. Subsequently, the migrated PMNs release neutrophil extracellular traps (NETs) to capture and kill invading pathogens [3,4].

The chemokine stromal cell-derived factor (SDF)-1 and its two receptors CXCR4 and CXCR7 are expressed widely in hematopoietic and non-hematopoietic cells [5,6]. Both SDF-1-receptors drive endothelial and epithelial migration of leukocytes during acute inflammation [6,7,8]. Further, CXCR4 seems to regulate the expression of adhesion molecules on platelets and neutrophils during acute inflammation [6,9]. CXCR7, also known as atypical chemokine receptor 3 (ACKR3), has been shown to play a crucial role for platelet activation and apoptosis during the acute inflammatory response [8,10]. In our previous study, we have already shown that inhibition of both SDF-1 receptors stabilizes the endothelial and epithelial integrity in acute inflammation via a functional adenosine receptor A2B (Adora2b) [7]. The extracellular nucleotide adenosine exerts its function through four purinergic adenosine receptors [11]. The G protein-coupled adenosine receptors A1, A2A, A2B, and A3 are involved in various inflammatory diseases [12,13,14]. Adora2b plays a predominant role in the migratory behavior of PMNs during acute inflammation [13,15,16,17].

Under physiological conditions, healthy glycocalyx and anti-coagulant molecules in the endothelium avoid the activation of adhesion of platelets and PMNs on the endothelium [18,19]. During sepsis, activated endothelial cells release pro-inflammatory and pro-coagulant molecules [20], supporting the activation of circulating platelets and PMNs [21,22]. Activated platelets search for activated PMNs to form PNCs [23]. PNCs promote the transmigration of PMNs into the inflamed tissue and play a crucial role during acute inflammation [24]. The Adora2b signaling path seems to regulate the interaction of platelets and neutrophils during an inflammatory response [25].

Bringmann et al., described a new mechanism of how PMNs trap and eliminate pathogens called NETs [26]. After inflammatory stimulation, PMNs extrude chromatin fibers decorated by antimicrobial peptides, such as neutrophil elastase (NE) and myeloperoxidase (MPO), to potentiate the antimicrobial effects of the NETs [27]. NETs allow PMNs to capture and kill pathogens by the release of anti-microbial peptides. An aggravated NET formation leads to excessive tissue damage and increased organ dysfunction [28]. New data suggest that the CXCR4 receptor in neutrophils is required to form NETs during bacterial infection or high-mobility group box 1 (HMGB1)-related cell death [29]. A recent work also suggested that the adenosine signaling is involved in NET formation [30].

Previous studies demonstrated that specific modulation of the PNC or NET formation may limit the tissue damage and be beneficial for organ function [28,31]. Recently, experimental data suggested protective effects of CXCR4 and CXCR7 antagonism during pulmonary inflammation and provided evidence for a link between the SDF-1-CXCR4/CXCR7 axis and adenosine receptor signaling [6,32].

In the present study, we investigated the influence of both SDF-1 receptors on PNC and NET formation during acute inflammation. Further, we evaluated the effects of specific CXCR4 and CXCR7 inhibition on both mechanisms and the link to a functional Adora2b.

## 2. Results

### 2.1. Expression of CXCR4 and CXCR7 in Platelets, Neutrophils, and Platelet–Neutrophil Complexes In Vivo and In Vitro

**Figure 1:**
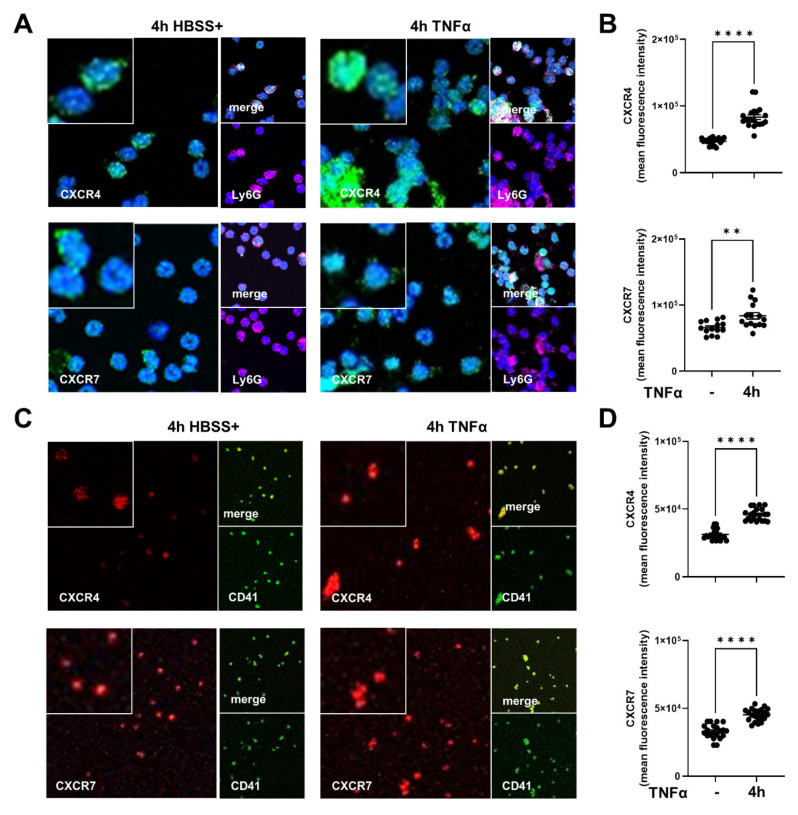

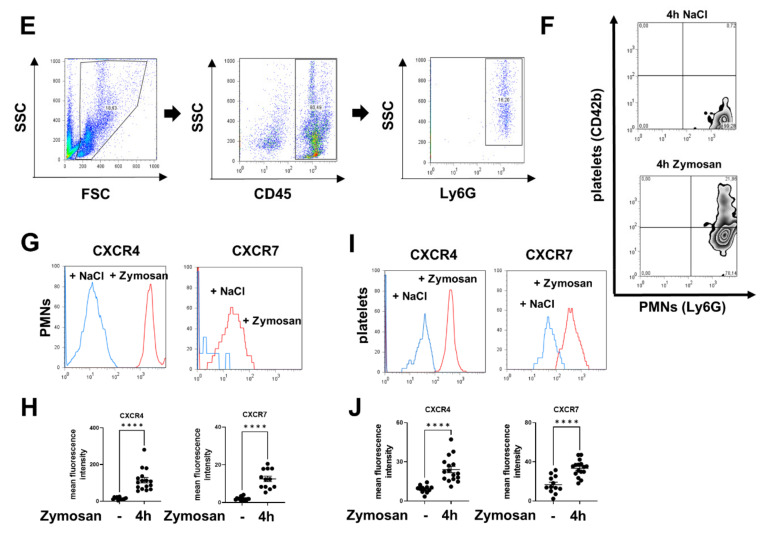
CXCR4 and CXCR7 expression in platelets, neutrophils, and platelet–neutrophil complexes (PNCs) during acute inflammation. CXCR4 and CXCR7 positive platelets and polymorphonuclear neutrophils (PMNs) were detected and quantified using flow cytometry and immunofluorescence staining. (**A**) Identifying human CXCR4 and CXCR7 positive (green) neutrophils (Ly6G; magenta) without activation (Hank’s buffered salt solution with Ca^2+^/Mg^2+^; HBSS+) and four hours after TNFα stimulation by immunofluorescence (images are representative slides of *n* = three independent experiments; 63× magnification). Nuclei counterstaining was performed using DAPI and appears blue. (**B**) Mean fluorescence intensity (MFI) of CXCR4 and CXCR7 in PMNs was quantified using ImageJ (*n* = 15–20). (**C**) Visualization of the surface expression of CXCR4 and CXCR7 (red) in human platelets (CD41; green) without and with TNFα administration (four hours) was performed by immunofluorescence (images are representative slides of *n* = three independent experiments; 63× magnification). (**D**) Quantification of CXCR4 and CXCR7 fluorescence intensity in human platelets by ImageJ (*n* = 20). (**E**) Leukocytes and PMNs in blood samples were identified by a flow cytometry method. The leukocyte gate was defined by the typical appearance of leukocytes in the forward scatter/side scatter (FSC/SSC plot). Then, CD45 positive cells were identified as leukocytes and PMNs were marked by a Ly6G antibody. (**F**) Illustration of PNC gating in blood samples with or without zymosan stimulation. Ly6G positive cells were detected as PMNs and CD42b (platelet surface marker) positive PMNs were identified as PNCs. (**G**) Representative histograms and (**H**) mean fluorescence intensity (MFI) of CXCR4 and CXCR7 expression in PNC-related PMNs were evaluated four hours after saline injection (blue histogram) or four hours after zymosan administration (red histogram) (*n* = 8–16). (**I**) CXCR4 and CXCR7 expression in murine platelets four hours after saline (blue histogram) and four hours after zymosan exposure (red histogram), respectively, displayed as histograms. (**J**) MFI of CXCR4 and CXCR7 in PNC-related platelets was determined by flow cytometry (*n* = 8–16). Data are presented as mean ± SEM; ** *p* < 0.01; **** *p* < 0.0001. Statistical analyses were performed by unpaired *t*-tests or Mann–Whitney tests.

First, we determined the expression of the SDF-1 receptors CXCR4 and CXCR7 in neutrophils, platelets, and platelet–neutrophil complexes with and without inflammation. We visualized the expression of CXCR4 and CXCR7 in freshly isolated PMNs from wild-type animals by immunofluorescence (Figure 1A) and quantified the mean fluorescence intensity (Figure 1B). TNFα stimulation significantly raised the expression of CXCR4 and CXCR7 in PMNs. Similarly, a higher surface expression of CXCR4 and CXCR7 in murine platelets was provoked by TNFα (Figure 1C,D). Next, we detected CXCR4 and CXCR7 expression in platelet–neutrophil complexes (PNCs) in blood samples. Therefore, we used a flow cytometry-based method to identify leukocytes and subsequently PMNs by a Ly6G positive signal as described before (Figure 1E) [33]. PMNs with an additional CD42b positive signal were determined as PNCs (right upper square of the dot plot) were raised in blood samples after zymosan (Figure 1F). To verify the role of both SDF-1 receptors in the formation of PNCs, we evaluated the expression of CXCR4 (Figure 1G) and CXCR7 (Figure 1H) in PNC-related PMNs and platelets, four hours after zymosan by flow cytometry. We observed an elevated CXCR4 and CXCR7 expression in PNC-associated PMNs (Figure 1I) and platelets (Figure 1J) in murine blood samples.

### 2.2. CXCR4 and CXCR7 Inhibition Ameliorates the Formation of Platelet–Neutrophil Complexes during Acute Inflammation

**Figure 2:**
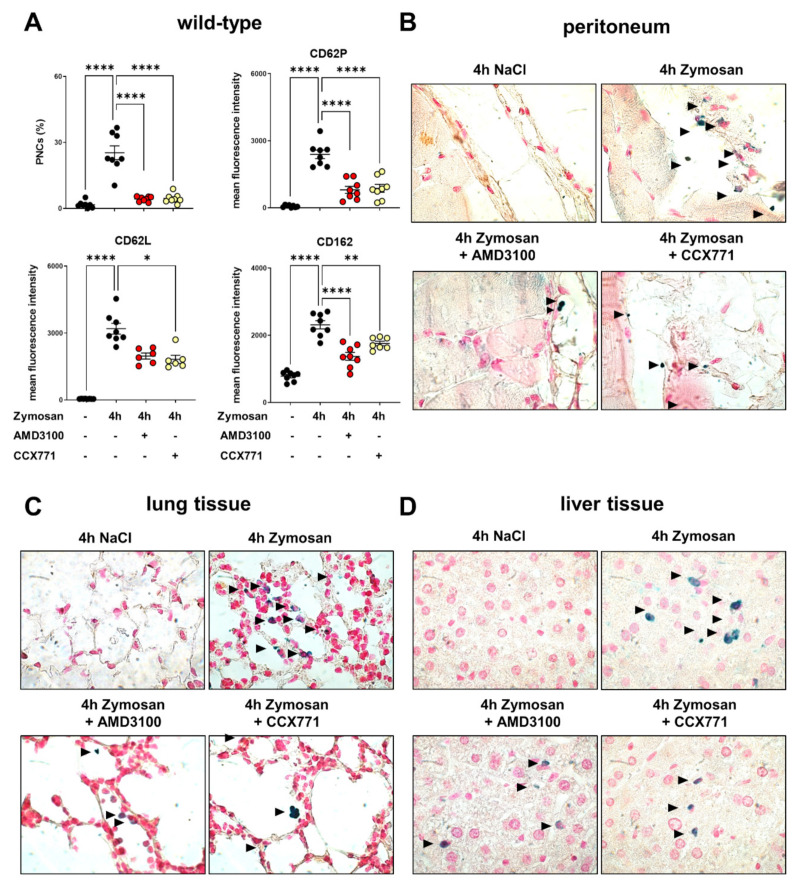

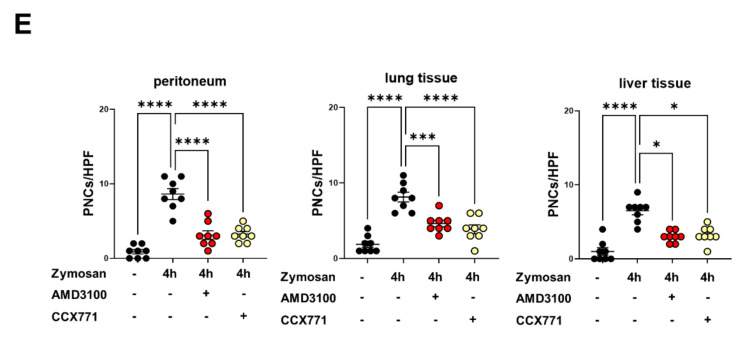
Influence of CXCR4 and CXCR7 inhibition in platelet–neutrophil complex (PNC) formation and expression of PNC-related molecules in wild-type animals. (**A**) PNC formation and PNC−related adhesion molecules in the blood samples of wild-type mice (black circles) with and without zymosan administration (*n* = 8-12 mice per group). Effects of CXCR4 (AMD3100; red circles) and CXCR7 (CCX771; yellow circles) inhibition on PNC formation and on the surface expression of PNC-related selectins CD62P, CD162, and CD62L. (**B**) PNC sequestration into the peritoneal tissue, (**C**) lung, and (**D**) liver tissue was evaluated by immunohistochemical staining. Polymorphonuclear neutrophils (PMNs) and platelets were tackled with specific antibodies, so that PMNs appeared blue and PNCs appeared blue/black (black arrows) (images are representative slides of *n* = three slides from each animal; *n* = four mice per group; 63× magnification). (**E**) PNC counts were enumerated by light microscopy. PNCs from three representative high-power fields were counted from three different slides (*n* = four mice per group). Data are presented as mean ± SEM; * *p* < 0.05; ** *p* < 0.01; *** *p* < 0.001; **** *p* < 0.0001. Multiple group comparison was analyzed by one-way ANOVA and Bonferroni correction or Kruskal–Wallis test.

Platelets scan for activated PMNs, form PNCs, and initiate neutrophil transmigration into the inflamed tissue [24]. In wild-type mice, zymosan administration raised PNC counts in blood samples. To evaluate the impact of the SDF-1 receptors CXCR4 and CXCR7, we used a selective CXCR4 (AMD3100) and selective CXCR7 antagonist (CCX771). Treatment with CCX771 or AMD3100 significantly reduced PNC formation in the blood of wild-type animals (Figure 2A). Further, we evaluated the surface expression of CD62P (also known as P-selectin), CD62L (also known as L-selectin), and CD162 (also known as P-selectin glycoprotein ligand-1) in PNCs. Selectins belong to the family of cell adhesion molecules and are involved in various adhesion and migratory processes [34]. Previous studies described the relevance of these selectins in the formation of PNCs during inflammation [24]. Four hours after zymosan administration, CD62P, CD62L, and CD162 expression was significantly raised in circulating PNCs. CXCR4 inhibition significantly decreased the expression of CD62P and CD162 in PNCs (Figure 2A). Moreover, the expression of CD62P, CD62L, and CD162 were significantly lower after CXCR7 inhibition (Figure 2A). To verify our flow cytometry data, PNC sequestration into the peritoneum (Figure 2B), lung (Figure 2C), and liver tissue (Figure 2D) was determined in immunohistochemical sections, where PMNs appear blue and PNCs appear blue/black. PNC counts in these slides were quantified. After zymosan, PNC counts increased in all tissues compared to those of untreated animals. The specific CXCR4 and CXCR7 antagonism decreased the PNC infiltration into all related tissues and confirmed our flow cytometry data (Figure 2E).

### 2.3. Adora2b Signaling Is Crucial for the Pharmacological Effects of CXCR4 and CXCR7 Antagonism on Inhibiting PNC Formation

**Figure 3:**
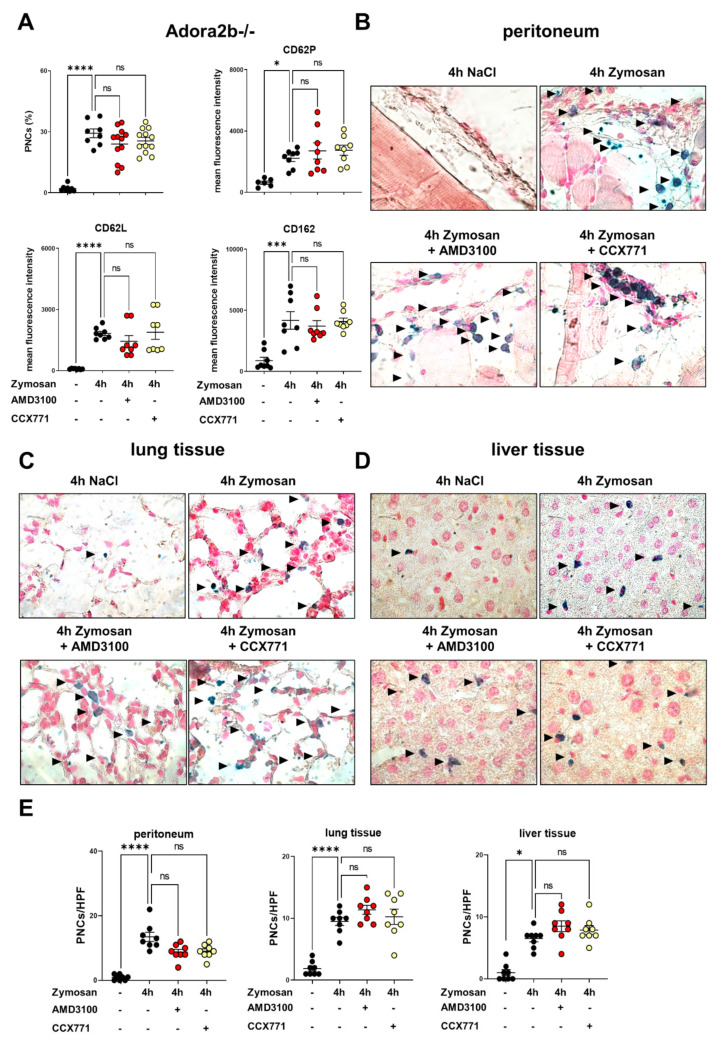
Effects of Adora2b depletion on platelet−neutrophil complex (PNC) formation and pharmacological inhibition of CXCR4 and CXCR7 during zymosan-induced peritonitis. (**A**) PNC formation and PNC-related adhesion molecules in the blood samples of Adora2b−/− mice (black circles) with and without zymosan administration (*n* = 8−12 mice per group). Effects of CXCR4 (AMD3100; red circles) and CXCR7 (CCX771; yellow circles) inhibition on PNC formation and on surface expression of CD62P, CD162, and CD62L. (**B**) The extravasation of PNCs in the peritoneal tissue, (**C**) lung, and (**D**) liver tissue slides was determined by immunohistochemistry. Polymorphonuclear neutrophils (PMNs) and platelets were tackled with specific antibodies, so that PMNs appeared blue and PNCs appeared blue/black (black arrows) (images are representative slides of *n* = three slides from each animal; *n* = four mice per group; 63× magnification). (**E**) PNC counts were enumerated by light microscopy. PNCs from three representative high-power fields were counted from three different slides (*n* = four mice per group). Data are presented as mean ± SEM; * *p* < 0.05; *** *p* < 0.001; **** *p* < 0.0001, ns: not significant. Multiple group comparison was analyzed by one-way ANOVA and Bonferroni correction or Kruskal–Wallis test.

Following previous studies and our hypothesis that the protective effects of CXCR4- and CXCR7 antagonism are linked with Adora2b signaling [6], we performed the same experimental setting with Adora2b−/− animals. Zymosan administration induced the formation of PNC counts and all PNC-related adhesion molecules in the blood of Adora2b−/− mice. The genetic Adora2b depletion abolished the anti-inflammatory effects of AMD3100 and CCX771 on PNC formation and PNC-related molecules (Figure 3A). To visualize our flow cytometry data, we also performed immunohistochemistry experiments with peritoneum, lung, and liver tissue slides from Adora2b−/− animals. Zymosan administration enhanced PNC formation in the peritoneum (Figure 3B), lung (Figure 3C), and liver tissues (Figure 3D) of Adora2b−/− mice compared to those of unstimulated animals, whereas the inhibition of CXCR4/CXCR7 did not lead to any changes. To quantify the PNC migration, PNCs were counted per high-power field and CCX771 or AMD3100 did not affect the zymosan-induced PNC influx into the peritoneal cavity, lung, and liver tissue, confirming our flow cytometry data (Figure 3E). These data highlight the link between the protective effects of CXCR4 and CXCR7 antagonism and a functional Adora2b signaling during acute inflammation.

### 2.4. Inhibition of CXCR4 and CXCR7 Dampens PNC Formation during Polymicrobial Inflammation

**Figure 4:**
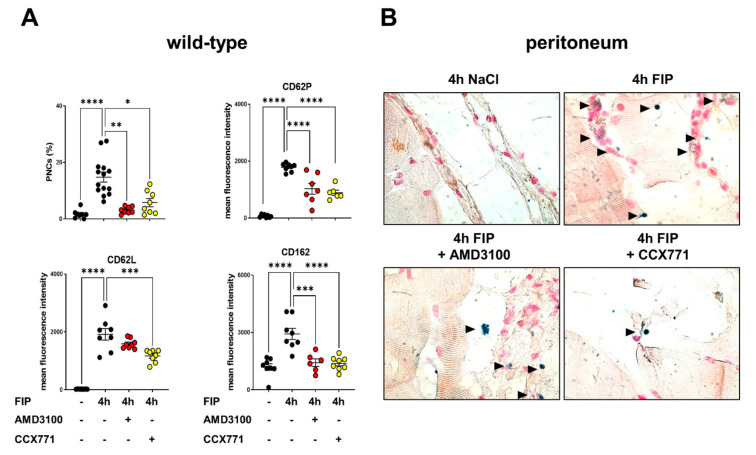

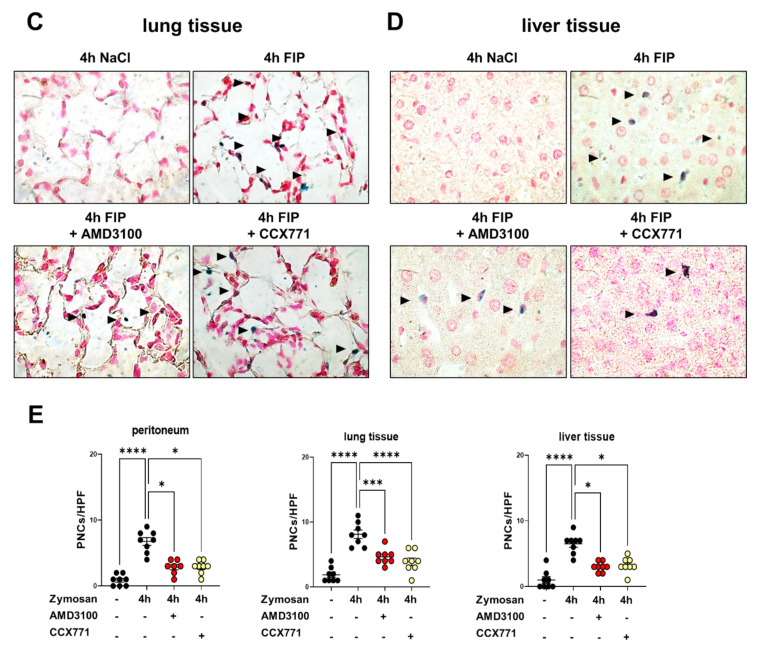
Role of CXCR4 and CXCR7 inhibition in platelet−neutrophil complex (PNC) formation during fecal-induced peritonitis and sepsis in wild-type animals. (**A**) Polymicrobial-induced PNC formation and expression of PNC−related molecules in the blood of wild-type mice (black circles) with and without fecal injection (*n* = 8 mice per group). CXCR4 (AMD3100; red circles) and CXCR7 (CCX771; yellow circles) inhibition ameliorated PNC formation and CD62P, CD162, and CD62L surface expression. (**B**) PNC migration into the peritoneal tissue, (**C**) lung, and (**D**) liver tissue was evaluated by immunohistochemical staining. Polymorphonuclear neutrophils (PMNs) and platelets were tackled with specific antibodies, so that PMNs appeared blue and PNCs appeared blue/black (black arrows) (images are representative slides of *n* = three slides from each animal; *n* = four mice per group; 63× magnification). (**E**) PNC infiltration was quantified by light microscopy. PNC formation from three representative high-power fields were counted from three different slides (*n* = three mice per group). Data are presented as mean ± SEM; * *p* < 0.05; ** *p* < 0.01; *** *p* < 0.001; **** *p* < 0.0001. Multiple group comparison was analyzed by one-way ANOVA and Bonferroni correction or Kruskal–Wallis test.

Next, we sought to verify the protective properties of specific CXCR4 and CXCR7 inhibition in a polymicrobial model of peritonitis and peritonitis-related sepsis [7]. The PNC formation in whole blood samples of wild-type animals four hours after the fecal instillation was determined by a flow cytometry-based method. Polymicrobial peritonitis increased PNC formation in blood samples compared to that of the unstimulated animals (Figure 4A). AMD3100 and CCX771 treatment significantly reduced PNC formation in blood samples and additionally, all PNC-related selectins were also affected after the inhibition of CXCR7. AMD3100 ameliorated the expression of CD62P and CD162, but not the PNC surface presence of CD62L. Subsequently, we performed the histological examination of the peritoneal (Figure 4B), lung (Figure 4C), and liver tissue (Figure 4D). PNCs were marked specifically and appeared blue/black (Figure 4B–D). We observed an increased PNC sequestration per high-power field into all tissues four hours after injecting the polymicrobial solution, which was abolished by the pharmacological inhibition of CXCR4 and CXCR7 (Figure 4E).

### 2.5. Specific CXCR4 and CXCR7 Inhibition Influences Intracellular Pathways and NETosis-Related Gene Expression

The activation of downstream signaling pathways and proteins regulates the expression of inflammation-related adhesion molecules and immune processes like NETosis [35]. To understand the protective effects of CXCR4 and CXCR7 inhibition at the intracellular level, we examined various intracellular signaling proteins. We evaluated mitogen-activated protein kinase 1 (MAPK1), MAPK3, rapidly accelerated fibrosarcoma 1 (RAF1), and the receptor for advanced glycation end products (RAGE) also known as AGER (advanced glycosylation and product-specific receptor), which all regulate the expression of inflammation-related selectins. Further, we examined the expression of arginine 1 (ARG1), lipocalin 2 (LCN2), S100 calcium-binding protein A8 (S100A8), myeloperoxidase (MPO), neutrophil elastase (NE), and peptidyl arginine deiminase type 4 (PADI4), which control NET formation during acute inflammation. Especially MPO, NE, and PADI4 are directly involved in NETosis [36].

**Figure 5:**
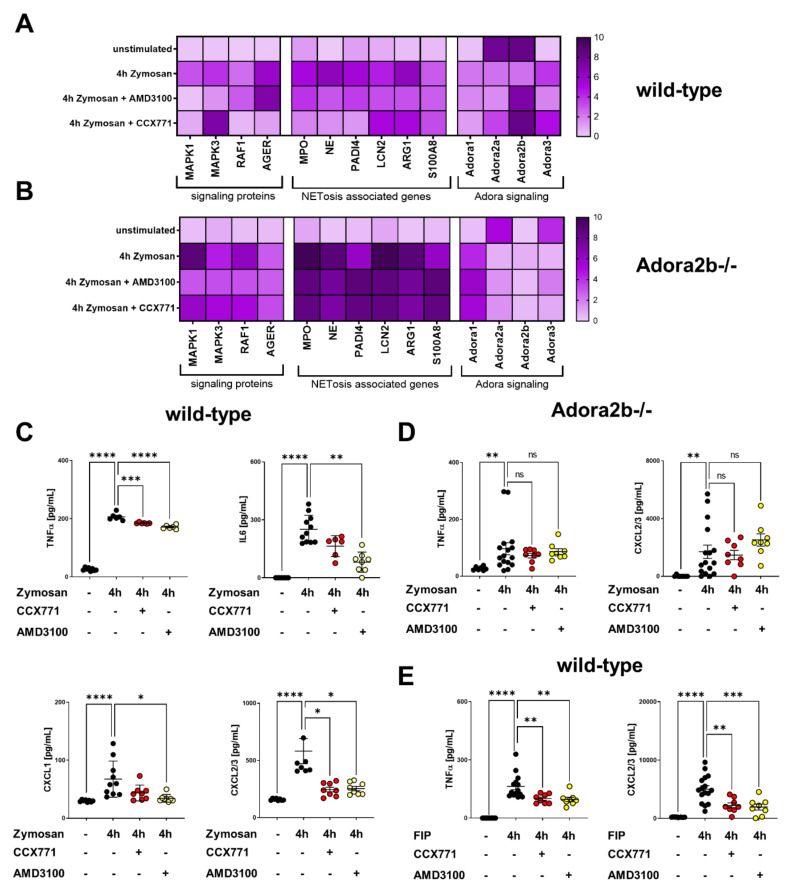
Inflammation and neutrophil extracellular traps (NET)-related gene expression and the release of inflammatory mediators. (**A**) The effects of specific CXCR4 and CXCR7 antagonism on the expression of different signaling pathways in polymorphonuclear neutrophils (PMNs) of wild-type and (**B**) Adora2b−/− mice are shown. Each row represents the normalized gene expression, each vertical column displays one gene, and each horizontal column illustrates a treatment group. Dark purple (upregulated) to light purple (unchanged) displays the level of gene expression (*n* = 6–8). (**C**) The release of TNFα, IL6, CXCL1, and CXCL2/3 into the plasma of wild-type mice in indicated conditions was measured without stimulation (black circles) and four hours after zymosan administration (black circles). Further, we evaluated the effects of CCX771 (red circles) and AMD3100 (yellow circles) on the zymosan-induced release of inflammatory mediators. (**D**) The plasmatic TNFα and CXCL2/3 release was measured in Adora2b−/− mice with and without zymosan stimulation (both black circles). Specific CXCR4 (AMD3100; yellow circles) and CXCR7 (CCX771; red circles) inhibition was evaluated in the plasma of Adora2b−/− four hours after zymosan stimulation. (**E**) The release of TNFα and CXCL2/3 four hours after fecal-induced peritonitis (FIP) in wild-type animals (black circles), AMD3100−treated (yellow circles) and CCX771−treated animals (red circles) (*n* = 6–12). Data are presented as mean ± SEM; * *p* < 0.05; ** *p* < 0.01; *** *p* < 0.001; **** *p* < 0.0001; ns: not significant. Multiple group comparison was analyzed by one-way ANOVA and Bonferroni correction or Kruskal–Wallis test.

Zymosan induced an elevation of the MAPK1, MAPK3, RAF1, and AGER gene expression in wild-type and Adora2b−/− animals compared to the corresponding control groups without stimulation (Figure 5A,B). AMD3100 reduced MAPK1 and MAPK3, but failed to affect RAF1 and AGER gene expression. The administration of CCX771 significantly attenuated MAPK1, RAF1, and AGER gene expression, whereas MAPK3 was not affected (Figure 5A). In Adora2b−/− animals, AMD3100 and CCX771 were unable to affect the gene expression of MAPK1, MAPK3, RAF1, and AGER (Figure 5B).

Further, zymosan administration resulted in an increased expression of all NET-related proteins in wild-type and Adora2b−/− mice (Figure 5A,B). The administration of AMD3100 led to a significant attenuation of MPO, PADI4, and S100A8. CCX771 demonstrated a significant reduction of MPO and PADI4 gene expression, but failed to reduce the expression of S100A8, LCN2, and ARG1 (Figure 5A). Neutrophils of Adora2b−/− animals presented increased ARG1, LCN2, S100A8, MPO, NE, and PADI4 gene expression after inflammation. AMD3100 and CCX771 administration failed to reduce the gene expression of all NET-related proteins and linked the protective effects of CXCR4 and CXCR7 inhibition with adenosine receptor Adora2b signaling (Figure 5B).

Previous studies provided evidence for a link between both SDF-1 receptors and Adora2b signaling [6]. Unstimulated wild-type animals had a high baseline of Adora2a and Adora2b gene levels. Four hours after stimulation, gene expression of Adora2a and Adora2b decreased. Pharmacologic inhibition of CXCR4 and CXCR7 increased the gene levels of Adora2b, but both antagonists failed to reverse the gene levels of Adora2a.

The activation of intracellular signaling proteins, such as MAPK1 and MAPK3, initiates the gene expression and the release of inflammatory mediators such as TNFα, IL6, CXCL1, and CXCL2/3 [37,38]. Zymosan induced the release of inflammatory cytokines and chemokines into the murine plasma compared to unstimulated animals. CXCR4 inhibition ameliorated the release of TNFα, IL6, CXCL1, and CXCL2/3. CCX771 showed protective effects on plasmatic levels of TNFα and CXCL2/3 (Figure 5C). The predominant effects of both antagonists were on the cytokine TNFα and the main neutrophil chemoattractant CXCL2/3. Therefore, we focused in our subsequent experiments on these two inflammatory mediators. After zymosan, Adora2b knockout animals had increased levels of TNFα and CXCL2/3. AMD3100 and CCX771 administration failed to improve the release of inflammatory cytokines in these animals, verifying our previous results (Figure 5D). We evaluated the plasmatic release of TNFα and CXCL2/3 in wild-type animals during polymicrobial sepsis to strengthen our findings. Both antagonists provided protective effects by reducing the release of inflammatory TNFα and CXCL2/3 in murine plasma after inflammation.

### 2.6. Effects of CXCR4 and CXCR7 Antagonism on Human PNC Formation and PNC Migratory Behavior

**Figure 6:**
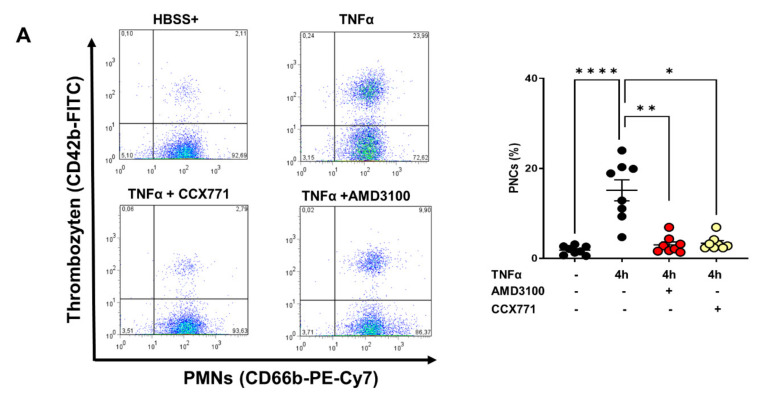

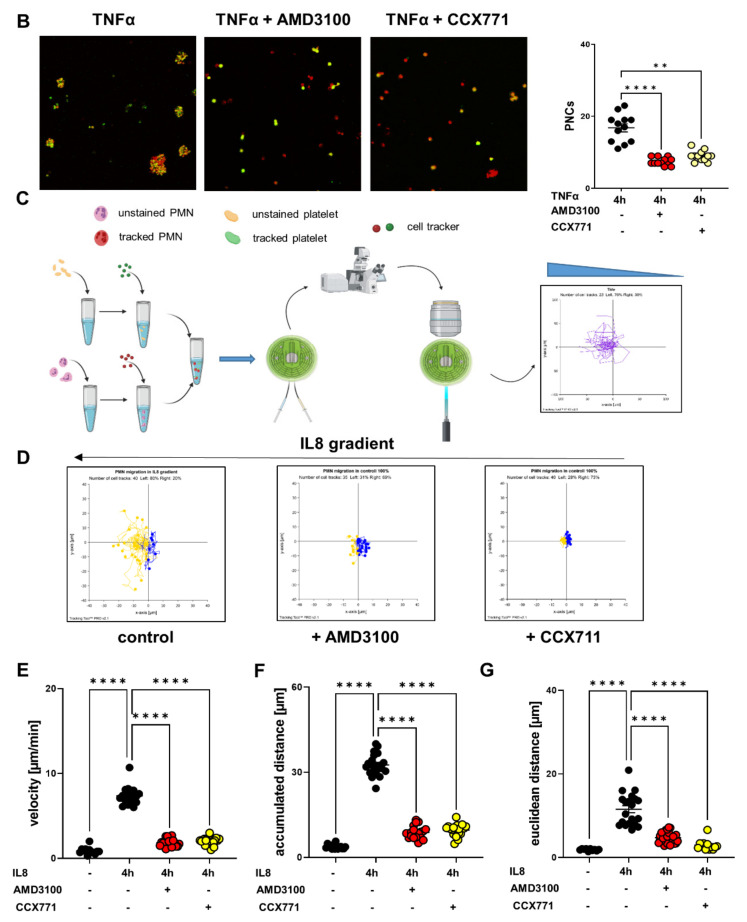
Effects of CXCR4 and CXCR7 blockade on formation of human platelet–neutrophil complexes (PNCs). (**A**) Freshly isolated PMNs and platelets were stimulated with TNFα and PNC formation was quantified using flow cytometry. PNCs were determined by a positive signal for CD66b and CD42b (right upper square in the dot plot). Comparison of PNC formation four hours after TNFα stimulation (black circles) and after specific CXCR4 (red circles) and CXCR7 inhibition (yellow circles) (*n* = 8–12). (**B**) Immunofluorescence staining of freshly isolated human PMNs (red) and platelets (green), and visualization of TNFα-induced PNC formation. Quantification of the PNC counts per visualization field and comparison of TNFα-stimulated PNCs (black circles) and after CXCR4 (red circles) and CXCR7 blockade (yellow circles) (*n* = 12). (**C**) Schematic illustration of the CellDirector^®^ staining method and migration assay. Fresh isolated human PMNs and platelets were tackled with red (PMNs) and green (platelets) cell tracker dyes. Subsequently, the stained cells were pooled and injected into the migration assay. The interleukin 8 (IL8)−related migratory behavior of the PNCs was observed by confocal microscopy. (**D**) Representative images of tracked PNCs’ migratory behavior upon an IL8 chemotactic gradient. Additionally, we quantified the influence of CXCR4 and CXCR7 blockade on IL8-related PNC migration. We also quantified the influence of CXCR4 and CXCR7 blockade on IL8-related PNC migration (*n* = 35–40 PNC counts). (**E**) Quantification of IL8-induced velocity, (**F**) accumulated distance, and (**G**) Euclidean distance of PNCs using the Tracking Tool™ Pro (Gradientech; Upsala; Sweden). Data are presented as mean ± SEM; * *p* < 0.05; ** *p* < 0.01; **** *p* < 0.0001. Multiple group comparison was analyzed by one-way ANOVA and Bonferroni correction or Kruskal–Wallis test.

To verify our in vivo findings, we isolated human platelets, i.e., PMNs from healthy volunteers, and evaluated PNC formation under inflammatory conditions by flow cytometry. TNFα stimulation led to a significant increase of PNC counts after four hours. CCX771 and AMD3100 treatment reduced PNC formation and confirmed our in vivo findings (Figure 6A). To verify and visualize these effects of inhibiting the SDF-1 receptors in human PNC formation, we performed immunofluorescence experiments. TNFα stimulation induced PNC formation. The treatment with AMD3100 and CCX711 ameliorated the PNC formation in our immunofluorescence experiments and confirmed our previous in vivo findings (Figure 6B).

We evaluated the effects of CXCR4 and CXCR7 inhibition on the migratory behavior of human PNCs using the CellDirector^®^ 2D chemotaxis assay. Human platelets and PMNs were isolated from healthy volunteers, and cell migration was performed (Figure 6C). More than 35 PNC counts were picked from each condition and tracked. The freshly formed PNCs followed the chemotactic IL8 gradient to the left side (marked yellow). Nevertheless, some PNCs moved in the opposite direction, which we interpreted as un-directional movement (shown in blue). According to our in vivo findings, specific CXCR4 and CXCR7 inhibition ameliorated PNC formation and impeded the migratory behavior of the PNCs (Figure 6D). Further, the velocity, accumulated distance, and Euclidean distance of the migrated PNCs were measured. The velocity represents the rolling speed of PNCs in the direction of the chemotactic gradient over ten minutes. The Euclidean distance represents the direct distance between the starting point and endpoint of the migrated PNCs. The total distance that the PNCs moved is defined by the accumulated distance. IL8 raised the PNC velocity, which was curtailed by AMD3100 and CCX771 administration (Figure 6E). Further, CXCR4 and CXCR7 inhibition limited the range of PNCs’ movement, displayed as the accumulated (Figure 6F) and Euclidean distance (Figure 6G), confirming the protective effects of AMD3100 and CCX771 treatment on PNC formation.

### 2.7. CXCR4 and CXCR7 Antagonism Affects the Formation of NETs Ex Vivo

**Figure 7:**
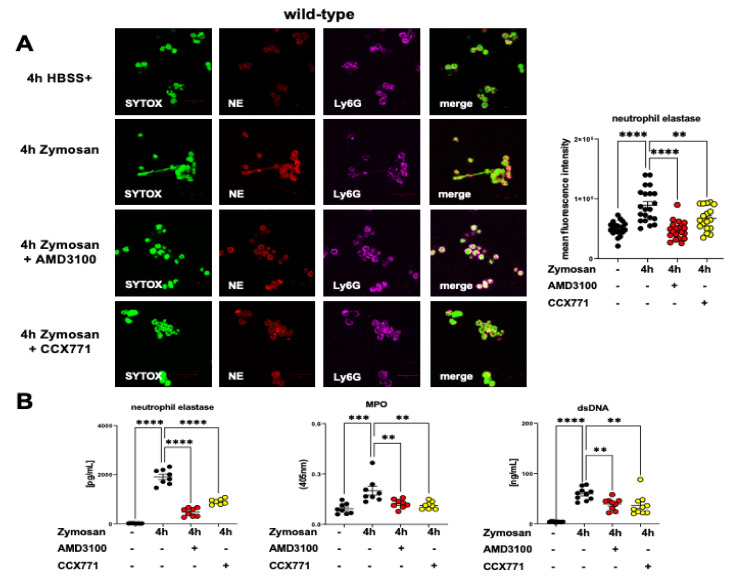

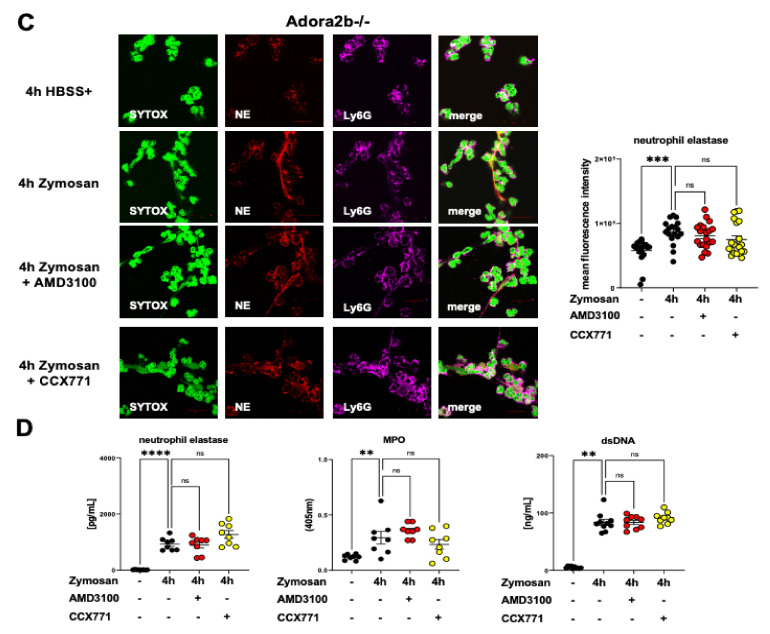
Effects of CXCR4 and CXCR7 inhibition on neutrophil extracellular trap (NET) formation during acute inflammation on wild-type and Adora2b−/− neutrophils. (**A**) Freshly isolated bone marrow PMNs from wild-type and (**C**) Adora2b−/− animals were stimulated for four hours with zymosan (100 ng) or HBSS+. The effects of AMD3100 and CCX771 treatment on NETosis on wild-type PMNs were evaluated by immunofluorescence experiments. Intracellular and extracellular DNA was stained by SYTOX (green). Neutrophil elastase (NE) (red) was evaluated as a marker for NETosis, and neutrophils (magenta) were marked by a specific Ly6G antibody (63x magnification; representative images from three independent experiments). Fluorescence intensity of NE in PMNs was quantified using ImageJ (*n* = 15–20). (**B**) The myeloperoxidase (MPO), dsDNA, and NE release from wild-type and (**D**) Adora2b−/− PMNs were evaluated by ELISA with or without stimulation (black circles) and the effects of AMD3100 (yellow circles) and CCX771 (red circles) treatment. Data are presented as mean ± SEM; ** *p* < 0.01; *** *p* < 0.001; **** *p* < 0.0001; ns: not significant. Multiple group comparison was analyzed by one-way ANOVA and Bonferroni correction or Kruskal–Wallis test.

The externalization of nuclear chromatin by neutrophils to trap invading pathogens is a crucial innate immune mechanism called NETosis [39]. Extranuclear chromatin fibers’ meshwork is decorated with neutrophil-derived proteases and enzymes such as neutrophil elastase and MPO [40]. At first, we evaluated the NET formation of freshly isolated murine PMNs by immunofluorescence experiments. We used SYTOX for the visualization of intracellular and extracellular chromatin. Zymosan stimulation activated the murine PMNs from wild-type animals and led to a raised expression of NE. The CCX771 and AMD3100 treatment significantly decreased the NE expression of PMNs from wild-type mice (Figure 7A). In additional ex vivo murine experiments, we determined the release of myeloperoxidase (MPO), double-stranded (ds)DNA, and NE from wild-type PMNs at indicated conditions. We observed a raised release of MPO, dsDNA, and NE four hours after zymosan stimulation. After CXCR4 and CXCR7 inhibition, a reduced expression of MPO, dsDNA, and NE was detected (Figure 7B). To corroborate the link between the SDF-1 receptors and the Adora2b signaling, we repeated the experiments and evaluated the MPO, NE, and dsDNA release from neutrophils of Adora2b−/− ex vivo. We detected an elevated NE expression in the PMNs of Adora2b−/− mice (Figure 7C) and raised MPO, NE, and dsDNA liberation four hours after zymosan stimulation. The CXCR4 and CXCR7 inhibition did not suppress NETosis and the release of antimicrobial proteases, supporting our previous findings (Figure 7D).

### 2.8. Effects of CXCR4 and CXCR7 Inhibition on Human NET Formation In Vitro

**Figure 8:**
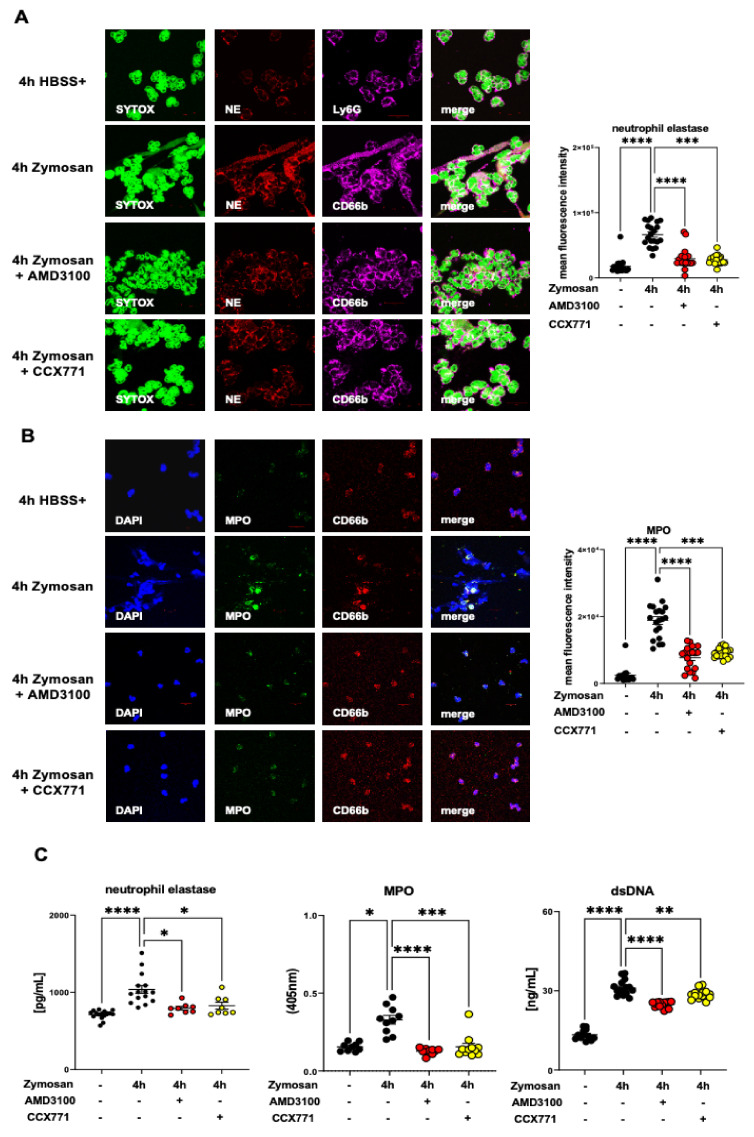
The role of specific CXCR4 and CXCR7 antagonisms in human neutrophil extracellular trap (NET) formation. (**A**) Fresh isolated polymorphonuclear neutrophils (PMNs) from healthy volunteers were stimulated for four hours with zymosan (100 ng) or HBSS+. The effects of CXCR4 and CXCR7 inhibition on NET formation were evaluated by immunofluorescence experiments. DNA marked by SYTOX (green), neutrophil elastase (NE) (red), and CD66b (magenta) as a PMN marker were stained by specific antibodies (63× magnification; *n* = three from three independent experiments). NE fluorescence intensity in human PMNs was assessed by ImageJ (*n* = 15–20). (**B**) Additionally, we evaluated the myeloperoxidase (MPO) activity (green) and DNA (DAPI; blue) in human PMNs (CD66b; red) without and with zymosan stimulation. The effects of AMD3100 and CCX771 on MPO activity were detected by immunofluorescence experiments (63× magnification; *n* = three from three independent experiments). Mean fluorescence intensity of MPO in human PMNs was evaluated by ImageJ (*n* = 15–20). (**C**) The MPO, dsDNA, and NE concentration in supernatant from human PMNs were evaluated in vitro by ELISA with or without zymosan stimulation (both black circles). The effects of AMD3100 (yellow circles) and CCX771 (red circles) treatment on the release of MPO, dsDNA, and neutrophil elastase was determined (*n* = 8−16). Data are presented as mean ± SEM; * *p* < 0.05; ** *p* < 0.01; *** *p* < 0.001; **** *p* < 0.0001. Multiple group comparison was analyzed by one-way ANOVA and Bonferroni correction or Kruskal–Wallis test.

To verify our murine findings, we isolated human PMNs and performed immunofluorescence experiments to evaluate the NET formation after zymosan administration. We observed a raised NE expression four hours after stimulation. AMD3100 or CCX771 treatment reduced the NET formation of human PMNs, confirming our murine data (Figure 8A). Further, we performed immunofluorescence experiments to detect the zymosan-induced MPO release from human PMNs. Both SDF-1 receptor inhibitors ameliorated the MPO presence in human PMNs and confirmed our previous findings (Figure 8B). The release of NE, MPO, and dsDNA in supernatants of our in vitro experiments with human PMNs was also elevated after zymosan stimulation. AMD3100 and CCX771 significantly diminished the expression of NE, MPO, and dsDNA after stimulation, confirming our results from murine ex vivo experiments (Figure 8C).

## 3. Discussion

The stromal cell-derived factor-1 (SDF-1) and its receptor CXCR4 are well-known to orchestrate the hematopoietic niche and regulate PMN release from bone marrow to vasculature during inflammation [41]. Activation of both SDF-1 receptors processes the canonical components of G-protein-coupled receptors and mediates activation of intracellular signals [42]. Additionally, CXCR4 and CXCR7 can operate as a heterodimer to regulate SDF-1-mediated functions [42]. Recently, CXCR4 and CXCR7 were identified as prominent chemokine receptors in thrombocytes [5,43]. However, the SDF-1 receptor CXCR4 is also highly expressed in neutrophils and in non-hematopoietic cells as well as CXCR7 [5,43]. Previous work demonstrated CXCR4 and CXCR7 under inflammatory conditions by focusing on atherosclerosis [9] and ischemic cardiac diseases [44,45]. Our data showed a high expression of both receptors in murine platelets and PMNs during acute peritoneal inflammation. In line with this data, current studies demonstrated an increase of CXCR4 and CXCR7 expression in platelets and PMNs after LPS stimulation, respectively TNFα activation [7,10,46].

Chemokines and chemokine receptors are involved in the interaction between platelets and neutrophils during acute inflammation [47,48]. Recently, Witte et al., demonstrated that the SDF-1 receptor CXCR4 is critically involved in the migration of activated platelets and interacts with leukocytes [49]. Further studies provided evidence for the regulatory role of both SDF-1 receptors in the behavior of neutrophils and platelets during various inflammatory models with consequences for the immune response [6,7,50,51]. Veenstra et al., showed that CXCR7 inhibition by CCX771 dampened leukocytes’ infiltration during brain inflammation [52]. Similarly, Kontos et al., demonstrated that a non-functional free-soluble CXCR4 receptor prevents leukocyte migration and thus reduces atherosclerotic inflammation [53]. However, neither study showed how the CXCR4 and CXCR7 blockade limited the migration of leukocytes and nor did they examine the interaction between leukocytes and platelets during acute inflammation. Consequently, we investigated the effects of CXCR4 and CXCR7 inhibition on PNC formation during sterile and polymicrobial sepsis. Our study showed that antagonism of CXCR4 and CXCR7 decreased PNC formation in the blood and ameliorated the extravasation of PNCs into the inflamed tissue. Here, selectins in PMNs and platelets are necessary to form PNCs [54]. The interaction between the platelet-related CD62P with the neutrophilic CD162 (also known as PSGL1) may represent the most important physical binding contact [24]. Previous studies demonstrated that a pharmacological blockade of CD62P or CD162 dampened PNC formation and PMN migration into the inflamed tissue [55,56].

Both adhesion molecules are critically involved in the formation of deep venous thrombosis, embolism, and atherosclerosis [57]. Therefore, direct CD62P or CD162 inhibition may negatively affect coagulation and induce bleeding [58]. Regarding this dilemma, possible therapeutic strategies can be an indirect inhibition of PNC-related selectins. PNCs and PNC-related selectins were dampened by specific CXCR4 and CXCR7 antagonism in both of our peritonitis models. In line with our murine data, the specific CXCR4 and CXCR7 inhibition ameliorated the inflammation-induced PNC formation of human platelets and PMNs in vitro.

Previous studies already identified extracellular adenosine as a protective mediator during hypoxia, ischemia, and reperfusion, and various inflammatory conditions [13,15,17,59,60,61]. Extracellular adenosine activates intracellular signaling pathways through four purinergic G-protein-coupled adenosine receptors (Adora1, Adora2a, Adora2b, and Adora3) [11,62]. Current data and own studies provide evidence for a link between the SDF-1-CXCR4/CXCR7 axis and Adora2b signaling [6,32]. In line with our previous projects, in the presented investigations, AMD3100 and CCX771 showed no protective effects if no functional Adora2b receptor was present.

NETosis plays a central part of the innate host defense but is regarded as “a double-edged sword” [39]. On the one hand, NETs immobilize and kill intruding pathogens, a crucial mechanism of pathogen clearance [26]. On the other hand, an enhanced NET formation during various acute and chronic disorders is associated with elevated tissue damage [63,64,65]. Previous studies suggested that the activation of CXCR4 in neutrophils mediates the formation of NETosis during chronic inflammation, such as in atherosclerosis and ischemic brain injury [66,67]. Radermecker et al., provided a link between the SDF-1 receptor CXR4 and NETs, and showed that CXCR4-positive PMNs are critically involved in the LPS-induced NET formation during chronic pulmonary inflammation [68]. Our data demonstrated that CXCR4 and CXCR7 inhibition diminished NETosis ex vivo and in vitro with murine and human PMNs. To the best of our knowledge, we are the first to describe the crucial role of the specific inhibition of SDF-1 receptors in NET formation during acute inflammation. Our data are in line with a recent study, which demonstrated that the activation of both SDF-1 receptors can potentiate NETosis during a chronic inflammatory process such as in cystic fibrosis [69].

It is well-known that the adenosine receptor signaling, especially the adenosine receptor A2A, is associated with NET formation [30,70]. Own studies provided evidence for a link between the SDF-1 receptors, CXCR4 and CXCR7, and the Adora2b signaling during acute inflammation [6,7]. To our knowledge, we are the first to describe that the protective effects of CXCR4 and CXCR7 inhibition on NETosis were associated with a functional Adora2b. In line with our data, Ramadan and colleagues demonstrated that adenosine agonism provided protective properties in autoimmune-induced NETosis [30].

AGER and the intracellular signaling proteins MAPK1, MAPK3, and RAF1 are necessary to initiate the transcription of inflammatory cytokines and chemokines during acute inflammation. Previous studies demonstrated that a specific inhibition of AGER, MAPK1, MAPK3, and RAF1 signaling reduced the expression of inflammatory cytokines and had beneficial effects during lethal sepsis [71,72]. These findings confirmed our current data, where AMD3100 and CCX771 ameliorated the expression of critical intracellular signaling proteins and inflammatory cytokines. Previously, we demonstrated that the selective CXCR4 and CXCR7 inhibition dampened the phosphorylation of the MAPK1 nuclear factor kappa-light-chain-enhancer of activated B cells (NF-κB) p65, both proteins controlling the transcription of genes, and subsequently reduced the liberation of inflammatory cytokines during acute peritoneal and acute pulmonary inflammation [6,7,8]. Our previous findings are consistent with our current observations, where AMD3100 and CCX771 significantly reduced the plasmatic concentrations of the critical inflammatory mediator TNFα and the potent neutrophilic chemoattractant CXCL2/3 [6,7,8].

In summary, our data revealed a previously uncharacterized role of the SDF-1 receptors CXCR4 and CXCR7 in activated platelets and PMNs during acute peritoneal inflammation. Specific inhibition of CXCR4 and CXCR7 dampened PNC formation and NETosis via functional Adora2b signaling. Our findings demonstrate a possible pharmacological target to regulate PNC and NET formation during sterile and polymicrobial peritonitis-related sepsis.

## 4. Material and Methods

### 4.1. Animals

Male C57BL/6J (Charles River; Germany) and Adora2b knockout mice (Adora2b−/−; a kind gift from Katya Ravid, D.Sc.; Boston University; School of Medicine, Department of Biochemistry; Boston, MA, USA) (8–14 weeks old; 20–25 g) were used for all experiments. The Animal Care and Use Committee of the University of Tübingen had approved all animal experiments.

### 4.2. Reagents

CCX771, a specific CXCR7 inhibitor (10 mg/kg body weight [BW]; subcutaneous injection [s.c.]; ChemoCentryx; San Carlos; CA, USA), and the specific CXCR4 inhibitor AMD3100 (10 mg/kg BW; intraperitoneal injection [i.p.]; Sigma-Aldrich; St. Louis; MO, USA) were administered one hour before zymosan application (Zymosan A of Saccharomyces cerevisiae; 50 mg/kg BW; i.p. injection; Sigma-Aldrich; St. Louis; USA).

### 4.3. Zymosan-Induced Peritonitis and Sepsis

Four hours after zymosan administration, three mL peritoneal lavage was retrieved after the injection of five mL phosphate-buffered solution without calcium (PBS-) into the peritoneal cavity. After thoracotomy, blood samples were collected by punctation of the right ventricle, and the vascular system was flushed by three mL PBS for blood-free organs. Peritoneum and organ (lung and liver) samples were removed and stored at −80 °C.

### 4.4. Fecal-Induced Peritonitis and Sepsis

To prepare the fecal solution, we collected dry fecal pellets randomly from C57BL/6J male mice cages with the same age and diet. Fecal material was pooled, diluted with normal saline to a concentration of 80 mg/mL, aliquoted, frozen, and the same fecal stock solution was used for this whole project. The fecal solution was injected intraperitoneally. After four hours, peritoneal lavage, blood, and organs were collected as described above.

### 4.5. Platelet–Neutrophil Complex Formation In Vivo and In Vitro

Zymosan was injected intraperitoneally in wild-type and Adora2b−/− mice, and murine blood samples were obtained four hours after the onset of the inflammation. We determined total leukocyte counts by light microscopy. The fraction of leukocytes, free circulating neutrophils, and platelet–neutrophil complexes were quantified by performing multicolor staining followed by flow cytometry. A typical leukocyte gate within the forward/sideward scatter (FSC/SSC) dot plot was created and the leukocyte population was defined by a CD45 peridinin-chlorophyll-protein (PerCP) (clone 30-F11; 103130; BioLegend; San Diego; CA, USA) positive staining. Then, we determined the PMNs by setting up a gate for Ly6G phycoerythrin/cyanin 7 (PE/Cy7) (clone 8A1; 127618; BioLegend; USA) staining in the SSC/Ly6G+ dot plot (Figure 1E). A fluorescein isothiocyanate (FITC)-conjugated CD42b antibody (clone Xia.G5; M040-1; Emfret; Würzburg; Germany) was used as specific platelet marker. For the differentiation between free-circulating PMNs and PNCs, we labeled the CD42b-expression in PMNs (clone 1A8; 127618; BioLegend; San Diego; USA) to detect the platelet–neutrophil complexes and separated them from CD42b negative PMNs by cross gating (Figure 1F). For the determination of the expression of PNC-related selectins in PNCs, we used an additional staining with CD62L pacific blue (clone MEL-14; 104424; BioLegend; San Diego; USA), CD62P allophycocyanin (APC) (clone RMP-1; 148304; BioLegend; San Diego; USA), and CD162 phycoerythrin (PE) (clone 2PH1; 555306; Becton Dickinson; Franklin Lakes; NJ, USA). Additionally, we evaluated the CXCR4 APC (clone 12G5; 306510; BioLegend; San Diego; USA) and CXCR7 PE (clone8F11-M16; 331104; BioLegend; San Diego; USA) expression in neutrophils and platelets in blood samples in different experiments.

Further, we obtained whole blood from healthy volunteers and isolated PMNs platelets. PMNs were isolated from citrated blood using a modified protocol of a Percoll-based density gradient as described before [73]. Whole blood and Percoll solutions were brought to room temperature. A gradient was prepared by pipetting 72% Percoll solution in the bottom of the tubes. After that, we added a middle layer with 63% Percoll solution. Whole blood was added carefully at the top of the layers. The gradient was centrifugated at room temperature for 30 min with 500 g with the brake turned off. After centrifugation, the PMN layer was located between the two Percoll solutions. PMNs were collected carefully and washed twice with cold HBSS without Ca^2+^/Mg^2+^ to avoid any prior activation. For the isolation of human platelets, we used a modified protocol as described before [74]. Citrated whole blood was centrifugated at 37 °C for 15 min with 150 g with the brake turned off. After centrifugation, we removed the supernatant with the platelet-rich plasma into a new tube and added acid-citrate-dextrane solution to avoid prior platelet aggregation. Next, we centrifugated the platelet-rich plasma at 37 °C and 400 g for five minutes. The platelet-rich pellet was resuspended with warm HBSS without Ca^2+^/Mg^2+^ to avoid any prior activation until the experiments started. PMNs and platelets were stimulated ex vivo for four hours with human recombinant tumor necrosis factor α (TNFα; 20 ng/mL; D63720; Promocell; Heidelberg; Germany) as described before [7]. Subsequently, PMNs and platelets were pooled, and the TNFα-induced formation of PNCs evaluated. We labeled the cell mix with a PE/Cy7-conjugated CD66b antibody to detect neutrophils (clone 139712; FAB946A; R&D Systems; Minneapolis; MN, USA) and FITC-conjugated CD42b antibody to determine platelets (clone 139712; FAB946A; R&D Systems; Minneapolis; USA). Samples were measured with the FACS Canto II flow cytometer (Becton Dickinson; Franklin Lakes; USA) and data analysis was performed using FlowJo software (Version 7.8.2; Ashland; Wilmington, DE, USA).

### 4.6. NET Quantification and Myeloperoxidase Release

To quantify the NET formation, we performed various complementary assays. We used the Quanti-iT double-stranded (ds) DNA PicoGreen assay kit (P7589; Invitrogen; Waltham; MA, USA) and murine neutrophil elastase (NE) (DY4517; R&D Systems; USA) ELISA to determine the release of extracellular dsDNA NE in the peritoneal lavage. Further, we evaluated the release of dsDNA (Quanti-iT dsDNA PicoGreen; P7589; Invitrogen; Waltham; USA) and human NE (DY9167; R&D Systems; Minneapolis; USA) in our in vitro experiments with human PMNs.

We determined the release of myeloperoxidase (MPO) as an activation marker for PMNs in the peritoneal lavage of mice four hours after zymosan and fecal administration. The MPO release from human PMNs from healthy volunteers was also determined at indicated conditions. MPO was measured colorimetrically at 405 nm by a plate reader (Tecan Reader Infinite M200PRO; Männedorf; Switzerland).

### 4.7. Immunohistochemical Detection of Platelet–Neutrophil Complexes

To detect PNCs, we performed immunohistochemical staining with paraffin-embedded peritoneal sections using the Vectastain ABC kit (PK-4000; Vector Laboratories; Burlingae; CA, USA). To prevent unspecific binding, the tissue sections were blocked for one hour with an avidin blocking solution (Vector Laboratories). Subsequently, sections were incubated with rabbit anti-mouse CD41 antibody (ab63983; Abcam; Cambridge; UK) to detect murine platelets overnight at 4 °C. Slides were incubated with biotinylated goat anti-rabbit IgG (BA-1000; Vector Laboratories; Germany) for one hour, followed by Vectastain ABC reagent for 30 min, and then developed by DAB substrate. Afterwards, PMNs were stained with rat anti-mouse Ly6G antibody (ab25377; Abcam; UK), followed by biotinylated rabbit anti-rat (BA-400; Vector Laboratories; Germany) and HistoGreen (E109; Linaris; Germany) as substrate. Counterstaining was performed using nuclear fast red (H-3403-500; Vector Laboratories; Germany). Rabbit IgG was used as control (31235; Invitrogen; USA) (Supplementary Figure S1A). The tissue slides were processed with a Leitz DM IRB microscope (Leica; Wetzlar; Germany) and analyzed with AxioVision (v4.8.2; Carl Zeiss; Jena; Germany). Sections were analyzed for the presence of PNCs (appear blue/black) by light microscopy and a manual count from three independent slides of each animal (*n* = three animals per group) at magnification x100 was performed.

### 4.8. Immunofluorescence Experiments

For in vitro immunofluorescence experiments, murine platelets and PMNs from whole blood were isolated by modified protocol, as described before [75,76] and incubated on chamber slides (Sarstedt Neumbrecht; Germany). Platelets and PMNs were stimulated for four hours with 100 ng/mL zymosan. After that, cells were fixed with 4% paraformaldehyde and after permeabilization with 1% Triton X-100, cells were blocked for one hour with 5% bovine serum albumin (BSA) in phosphate-buffered saline solution (PBS). Murine PMNs and platelets were labeled using rat monoclonal anti–Ly6G (sc19648; Santa Cruz Biotechnology; Dallas; TX, USA), rabbit polyclonal anti-CD41 (ab63983; Abcam; UK), goat polyclonal anti-CXCR7 (sc-107515; Santa Cruz Biotechnology; USA), and rabbit polyclonal anti-CXCR4 (sc-9046; Santa Cruz Biotechnology; USA). IgG controls are displayed in Supplementary Figure S1B,C. In additional experiments, we evaluated the expression of neutrophil elastase in murine PMNs from wild-type and Adora2b−/− animals four hours after zymosan stimulation and the effects of CXCR4- (AMD3100; 1µM; Sigma-Aldrich; USA) or CXCR7-inhibition (CCX771; 1 µM; ChemoCentryx; USA). Therefore, murine PMNs were stained using rat monoclonal anti-Ly6G (sc19648; Santa Cruz Biotechnology; USA), goat polyclonal anti-neutrophil elastase (Santa Cruz Biotechnology; USA), and SYTOX™ green nucleic acid stain (S7020; Invitrogen; USA). For visualization, the following secondary antibodies were employed: polyclonal donkey anti-goat IgG Alexa Fluor 488 (A11055; Thermo Fisher Scientific; Waltham; MA, USA), polyclonal goat anti-rabbit IgG Alexa Fluor488 (A11008; Thermo Fisher Scientific; Germany), and polyclonal rabbit anti-goat IgG Alexa Fluor 546 (A21085; Thermo Fisher Scientific; Germany). For nuclei counterstaining, we used Roti-Mount FluorCare DAPI (HP20.1; Carl Roth; Karlsruhe; Germany).

Further, human platelets and PMNs from citrated whole blood of healthy volunteers were isolated as described above in Section 4.5. We incubated the isolated platelets and PMNs for four hours with TNFα or with Hank´s balanced salt solution with Ca^2+^/Mg^2+^ (HBSS+). Additional groups were pretreated with the CXCR4- (AMD3100; 1 µM; Sigma-Aldrich) or CXCR7-antagonists (CCX771; 1 µM). Subsequently, cells were fixed with 4% paraformaldehyde, permeabilized with 1% Triton X-100, and blocked for one hour with 5% BSA/PBS solution. Human cells were stained using mouse monoclonal anti-CD15 (sc19648; Santa Cruz Biotechnology; USA), rabbit polyclonal anti-CD41 (ab63983; Abcam; UK), SYTOX™ green nucleic acid stain (S7020; Invitrogen; USA), goat polyclonal anti-MPO (AF3667; R&D Systems; USA), followed by the secondary antibodies as described above. IgG controls are displayed in Supplementary Figure S2A,B. Images were analyzed using ZEN software (Black edition 2011; Zeiss; Jena; Germany) and mean fluorescence intensities were measured using ImageJ (Version 1.49v; National Institute of Health; Bethesda; Rockville, MD, USA).

To evaluate the migratory behavior of human PNCs in response to the potent chemoattractant interleukin 8 (IL8), we used the cell-based assay CellDirector^®^ 2D (Gradientech™; Uppsala, Sweden). We isolated human PMNs and platelets from healthy volunteers and labeled neutrophils with a red CellTracker (CMTPX; C34552; Invitrogen; USA) and platelets with green CellTracker (CMFDA; C29025; Invitrogen; USA). PMNs and platelets were pooled, treated with AMD3100 (1 µM; Sigma-Aldrich; USA) respectively CCX771 (1 µM; ChemoCentryx; USA) or without any antagonist, and stimulated with zymosan (100 ng/mL; Sigma-Aldrich; USA). We injected the pooled PMNs and platelets into the CellDirector^®^ 2D assay, following the recommendations of the manufacturer. A chemotactic gradient was created in the central position of the CellDirector^®^ 2D assay by continuous infusion of human recombinant IL8. Images were recorded using ZEN software (Black edition 2011; Zeiss; Jena, Germany), and PNC tracking was performed using the TrackingTool™ PRO (Gradientech; Sweden). The TrackingTool™ PRO analyzed PNC velocity and the accumulated distance of the migrated PNCs. Further, we determined the direct smallest distance between the starting point and endpoint of the migrated PNCs, also known as Euclidean distance.

### 4.9. RT-PCR

Total RNA was isolated from murine PMNs using pegGOLD TriFast (Peqlab; Erlangen; Germany). cDNA synthesis was performed with Bio-Rad iScript kit (Bio-Rad; Munich; Germany) according to the manufacturer’s directions. We evaluated the expression of murine mitogen-activated protein kinase 1 (MAPK1), mitogen-activated protein kinase 3 (MAPK3), rapidly accelerated fibrosarcoma 1 (RAF1), advanced glycosylation end product-specific receptor (AGER), lipocalin 2 (LCN2), S100A8, myeloperoxidase (MPO), neutrophil elastase (NE), and peptidyl arginine deiminase type 4 (PADI4) by qRT-PCR, using the following primers: MAPK1 (5′-AAT TGG TCA GGA CAA GGG CTC-3′ and 5′-GAG TGG GTA AGC TGA GAC GG-3′), MAPK3 (5′-AGT CTC TGC CCT CGA AAA CC-3′ and 5′-ACT GTG ATG CGC TTG TTT GG-3′), RAF1 (5′-CGT GGA GAC GAG TGT CGA G-3′ and 5′-GAG CCA TCA AAC ACC GCA TC-3′), AGER (5′-GGT CAC AGA AAC CGG CGA TG-3′ and 5′-GCA TGG ATC ATG TGG GCT CT-3′), LCN2 (5′-CAC CAC GGA CTA CAA CCA G-3′ and 5′-CGT TCC TTC AGT TCA GGG G-3′), ARG1 (5′-GTG AAG AAC CCA CGG TCT G-3′ and 5′-GCA CCA CAC TGA CTC TTC C-3′), S100A8 (5′-GAC AAT GCC GTC TGA ACT GG-3′ and 5′-GCT ACT CCT TGT GGC TGT TT-3′), MPO (5′-GTG CCT TTG TAC GCT GGT TG-3′ and 5′-TGG GCC GGT ACT GAT TGT TC-3′), NE (5′-ACC CAG TGT GCT ACA AGA GC-3′ and 5′-GTG CAT ACG TTC ACA CGA CG-3′), and PADI4 (5′-ATA GCG GTT ACT CCA GCA GC-3′ and 5′-CCC GGT TCC AGT CGA TAC AG-3′). Additionally, we evaluated the expression of the adenosine receptors: Adora1, Adora2a, Adora2b, and Adora3 using the following primers: Adora1 (5′-ATT GTC ACT CAG CTC CCG C-3′ and 5′-TCA CCA GTA CAT TTC CGG GC-3′), Adora2a (5′-TCA ACA GCA ACC TGC AGA AC-3′ and 5′-GGC TGA AGA TGG AAC TCT GC-3′), Adora2b (5′-GCG TCC CGC TCA GGT ATA AA-3′ and 5′-CAG TGG AGG AAG GAC ACA CC-3′), and Adora3 (5′-GGG TTC CTG TAC TTC CTC TTG G-3′ and 5′-TCA ACC TCA GCC GCT TAT CC-3′). 18s was used as housekeeping gene (5′-´GTA ACC CGT TGA ACC CCA TT-3′ and 5′-CCA TCC AAT CGG TAG TAG CG-3′).

### 4.10. Cytokine and Chemokine Concentration

We determined the release of tumor necrosis factor α (TNFα), interleukin 6 (IL6), CXCL1, and CXCL2/3 in murine plasma of wild-type and Adora2b−/− mice four hours after zymosan- and fecal-administration by ELISA kits, following the manufacturer’s directions (DY406; DY453; DY452; DY410; R&D Systems; USA).

### 4.11. Software and Statistical Analysis

Data are presented as mean ± SEM unless indicated otherwise. The distribution was evaluated by investigating kurtosis, skewness, Q-Q plots, and histograms. Additionally, blinding of the experimenters took place in the animal experiments to avoid subjective bias. For comparisons between more than two groups, one analysis of variance (ANOVA) was performed and adjusted by Bonferroni correction. In the case of a not normal distribution, nonparametric methods were used (Kruskal–Wallis test). Student *t*-test was used to compare two groups, which were normally distributed, while Mann–Whitney tests were used to evaluate skewed variables. Statistical analysis was performed using GraphPad Prism for Windows (Version 9.1; GraphPad Software; San Diego; CA, USA); *p* < 0.05 was considered statistically significant. All schematic graphics were designed with BioRender.com (2021.09.06) and mean fluorescence intensities were measured by ImageJ (Version 1.49v; National Institute of Health; USA) respectively using FlowJo software (Version 7.8.2; USA).

## Data Availability

The datasets generated during and/or analyzed during the current study are available from the corresponding author on reasonable request.

## Supplementary Materials

The following are available online at https://www.mdpi.com/article/10.3390/ijms222413576/s1.

## Abbreviations

Adora2b	Adenosine receptor A2B
AGER	Advanced glycosylation end product-specific receptor
ACKR3	Atypical chemokine receptor 3
APC	Allophycocyanin
CXCL	C-X-C motif chemokine ligand
DAPI	4‘,6-diamidin-2-phenylindol
ELISA	Enzyme-linked immunosorbent assay
ERK1/2	Extracellular signal-regulated kinase 1/2
FITC	Fluorescein isothiocyanate
FSC	Forward scatter
HBSS	Hank´s balanced salt solution
HMGB1	High-mobility-group-box 1
IL	Interleukin
LCN2	Lipocalin 2
MAPK	Mitogen-activated protein kinase
MPO	Myeloperoxidase
NE	Neutrophil elastase
NET	Neutrophil extracellular traps
PADI4	Peptidyl arginine deiminase type 4
PE	Phycoerythrin
PE/Cy7	Phycoeryhtrin/cyanin 7
PerCP	Peridinin-chlorophyll-protein
PMNs	Polymorphonuclear neutrophils
PNCs	Platelet–neutrophil complexes
PBS-	Phosphate-buffered solution without calcium
RAF1	Rapidly accelerated fibrosarcoma 1
SDF-1	Stromal cell-derived factor-1
SSC	Sideward scatter
TNF-α	Tumor necrosis factor alpha
