# Supplementary material for: CXCR4 and CXCR7 Inhibition Ameliorates the Formation of Platelet–Neutrophil Complexes and Neutrophil Extracellular Traps through Adora2b Signaling
Source: Int J Mol Sci. 2021 Dec 17;22(24):13576. doi: 10.3390/ijms222413576 (PMC8709064; doi:10.3390/ijms222413576)
Supplement: Supplementary file 1 [file ijms-22-13576-s001.zip › ijms-1480620-supplementary.pdf]

Supplementary Figure S1

A

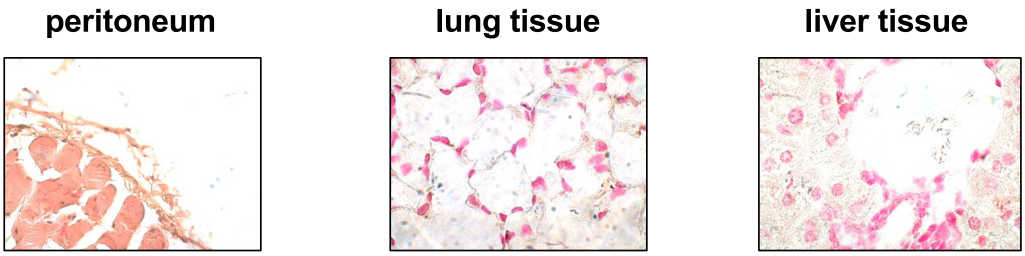

B

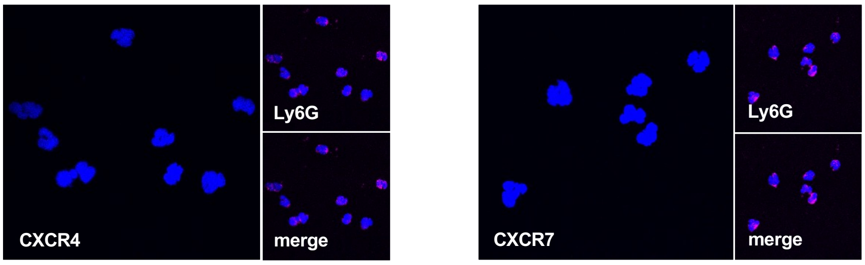

C

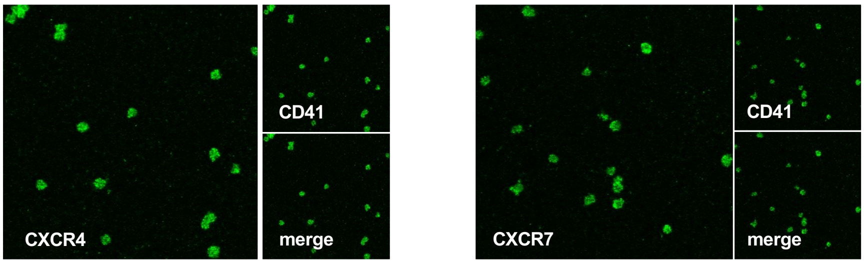

Supplementary Figure S1. (**A**) IgG controls of PNC immunohistochemical staining from the peritoneum, lung and liver tissue of wild-type animals (original magnification 100x; one representative image of four is shown; n=4). (**B**) Immunofluorescence experiments with unspecific IgG antibodies served as control staining for the detection CXCR4 and CXCR7 on PMNs and (**C**) platelets (original magnification 63x; one representative image of four is shown; n=4). PMNs were labeled by specific Ly6G antibody and appear magenta and DAPI was used as nuclear marker (blue). A CD41 antibody was used to mark platelets and appeared green.

Supplementary Figure S2

A

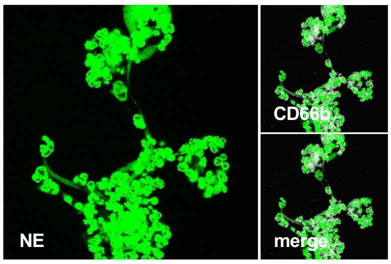

B

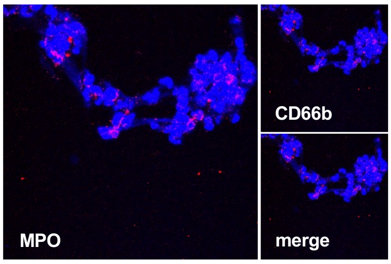

Supplementary Figure S2 (**A**) Unspecific IgG antibodies served as control staining for the detection of neutrophil elastase (NE) and (**B**) myeloperoxidase (MPO) on human PMNs. Human PMNs were labeled by specific CD66b antibody and appear magenta or red. As marker for deoxyribonucleic acid, we used SYTOX (green) or DAPI (blue)(original magnification 63x; one representative image of four is shown; n=4).
